# Histone H3 lysine K4 methylation and its role in learning and memory

**DOI:** 10.1186/s13072-018-0251-8

**Published:** 2019-01-07

**Authors:** Bridget E. Collins, Celeste B. Greer, Benjamin C. Coleman, J. David Sweatt

**Affiliations:** Department of Pharmacology, Vanderbilt University, 2220 Pierce Avenue, Nashville, TN 37232 USA

**Keywords:** Learning, Memory, Epigenetics, Neuroepigenetics, Histone methylation, H3K4

## Abstract

Epigenetic modifications such as histone methylation permit change in chromatin structure without accompanying change in the underlying genomic sequence. A number of studies in animal models have shown that dysregulation of various components of the epigenetic machinery causes cognitive deficits at the behavioral level, suggesting that proper epigenetic control is necessary for the fundamental processes of learning and memory. Histone H3 lysine K4 (H3K4) methylation comprises one component of such epigenetic control, and global levels of this mark are increased in the hippocampus during memory formation. Modifiers of H3K4 methylation are needed for memory formation, shown through animal studies, and many of the same modifiers are mutated in human cognitive diseases. Indeed, all of the known H3K4 methyltransferases and four of the known six H3K4 demethylases have been associated with impaired cognition in a neurologic or psychiatric disorder. Cognitive impairment in such patients often manifests as intellectual disability, consistent with a role for H3K4 methylation in learning and memory. As a modification quintessentially, but not exclusively, associated with transcriptional activity, H3K4 methylation provides unique insights into the regulatory complexity of writing, reading, and erasing chromatin marks within an activated neuron. The following review will discuss H3K4 methylation and connect it to transcriptional events required for learning and memory within the developed nervous system. This will include an initial discussion of the most recent advances in the developing methodology to analyze H3K4 methylation, namely mass spectrometry and deep sequencing, as well as how these methods can be applied to more deeply understand the biology of this mark in the brain. We will then introduce the core enzymatic machinery mediating addition and removal of H3K4 methylation marks and the resulting epigenetic signatures of these marks throughout the neuronal genome. We next foray into the brain, discussing changes in H3K4 methylation marks within the hippocampus during memory formation and retrieval, as well as the behavioral correlates of H3K4 methyltransferase deficiency in this region. Finally, we discuss the human cognitive diseases connected to each H3K4 methylation modulator and summarize advances in developing drugs to target them.

## Background

“Life is not what one lived, but what one remembers and how one remembers it in order to recount it.”

- *Gabriel García Márquez, Living to Tell the Tale (as translated by Edith Grossman, 2004)*

In the epigraph of his autobiography, Colombian novelist Gabriel García Márquez brings to literary light a fundamental consequence of neuroscience—that our memories subsume the reality of our past. In holding such perceived truth, memories provide a basis for human behavior. Memories allow us, for example, to forge lasting relationships, to reliably navigate our environment, to form a continuous scientific knowledgebase. Impairments in learning and memory, therefore, can have devastating consequences for an individual’s ability to function independently in society. Additionally, the persistence of undesirable memories, such as those of trauma or violence, can contribute to mental illness leading to social inhibition. Clearly, understanding the mechanisms of memory formation is of immense scientific interest as well as of great social and medical benefit.

In this pursuit, neuroepigenetics has emerged as an exciting subfield of neuroscience [1, 2]. Encompassing both developmental and experience-dependent epigenetic mechanisms, neuroepigenetics describes how chromatin modifications such as DNA methylation, histone posttranslational modifications (PTMs), and histone variant exchange permit the proper genesis and adaptability of neurons. Together, these modifications comprise a neuroepigenetic ensemble that exhibits learning-induced changes concurrent with the transcriptional changes required for synaptic structuring. Accordingly, neuroepigenetics establishes a plausible working model for how memories are encoded in neurons at the molecular level [3].

**Fig. 1:**
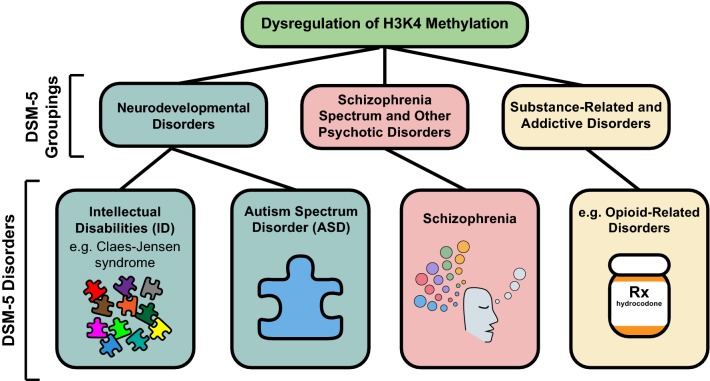
Cognitive diseases associated with dysregulation of H3K4 methylation. Associated diseases fall under three different DSM-5 groupings: Neurodevelopmental disorders, schizophrenia spectrum and other psychotic disorders, and substance-related and addictive disorders. Neurodevelopmental disorders encompass both ID and ASD. Beneath each DSM-5 disorder is an icon that either symbolizes the disorder or depicts a key clinical feature of it (Far left: A collection of puzzle pieces, representing the complexity of genetic and non-genetic etiologies of ID. Middle left: A blue puzzle piece, one of the symbols of ASD used by advocacy organizations. Middle right: A representation of visual and auditory hallucinations experienced by patients with schizophrenia. Far right: A prescription bottle of hydrocodone, representing one of the compounds used by patients with this disorder)

Lysine methylation of unstructured histone tails is being increasingly understood as an important component of this neuroepigenetic ensemble [4–11]. In particular, regulation of histone H3 lysine 4 (H3K4) methylation, widely regarded as a mark of transcriptional activation, has been implicated in both hippocampus (HPC) and striatum-dependent memory formation in mice; furthermore, mutations in many genes encoding H3K4 methylation modifiers are implicated in human cognitive dysfunction (Fig. 1) [12–19]. Given the prominence of H3K4 methylation and its relevance to transcriptional regulation in all cells, we focus our review on this specific epigenetic mark—its establishment and regulation, its role in cognition, and its dysregulation in disease.

## H3K4 methylation marks: from identification to genomic localization and applications to neuroscience

**Fig. 2:**
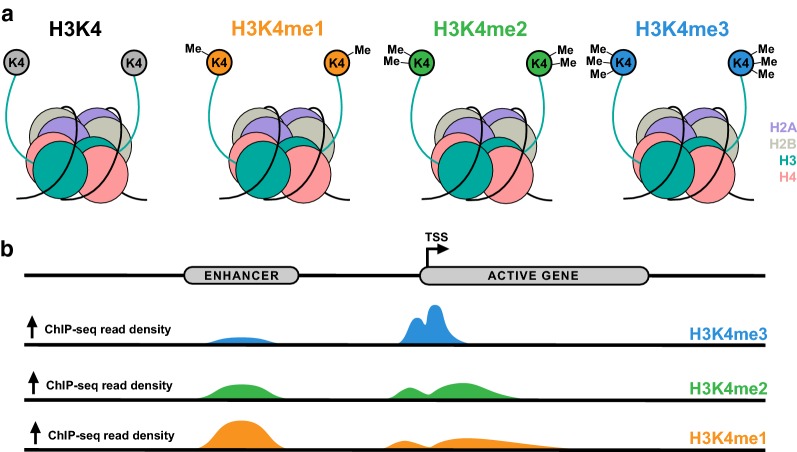
H3K4 methylation marks and their genomic distributions. **a** Histone octamers, comprised of two of each histone H2A, H2B, H3, and H4 subunits, are wrapped by DNA to form nucleosomes. An N-terminal tail protrudes from each histone subunit, containing residues amenable to posttranslational modification. Lysine residue K4 on H3 can be mono- (orange), di- (green), or tri- (blue) methylated, forming H3K4me1, H3K4me2, or H3K4me3, respectively. **b** H3K4me1, H3K4me2, and H3K4me3 are located in different regions throughout the genome. H3K4me3, H3K4me2, and H3K4me1 are found in increasingly broad distributions about the TSSs of actively transcribed genes. Additionally, H3K4me1 is found at distal regions acting as enhancers

Lysine methylation of histones was first described in 1964, and H3K4-specific methylation was first described one decade later in histones extracted from rainbow trout testes [20, 21]. Since then, H3K4 methylation has been shown to be a highly conserved chromatin modification, occurring in a variety of organisms from the ciliate protozoan *Tetrahymena*, to the simple eukaryote yeast, to humans [22]. H3K4 methylation proceeds in a graded fashion, producing mono-, di-, or trimethylated H3K4 (H3K4me1, H3K4me2, and H3K4me3, respectively) and, with unmethylated H3K4, generates a quaternary system of control (Fig. 2a).

Given this multiplicity of methylation, quantification of individual methylation states posed an early methodological challenge. Initial efforts relied on Edman degradation to determine methylation sites from histone protein sequences; however, this method’s time-intensive nature and requirement for large amounts of starting material caused it to fall out of favor as newer mass spectrometry and antibody-based approaches, such as Western blotting and chromatin immunoprecipitation (ChIP), became available [23, 24]. Combining these techniques with experimental designs incorporating model animals and behavioral tests has allowed a better understanding of the dynamic regulation of H3K4 methylation in the brain.

Using methylation state-specific antibodies, total levels of different H3K4 methylation states can be determined by Western blotting or enzyme-linked immunosorbent assays (ELISA). Additionally, ChIP has allowed the detection of genomic regions enriched in H3K4 methylation marks, or H3K4 methylation “peaks.” With quantification initially restricted to the use of site-specific primers, the field was limited to low-throughput probing for methylation level at single genomic loci. Later studies, however, were able to generate genome-wide maps of H3K4 methylation marks using technologies such as ChIP-chip, following ChIP with DNA microarrays, and ChIP-seq, following ChIP with massively parallel DNA sequencing [25–28]. ChIP-chip requires designing probes to a known set of genomic loci, whereas ChIP-seq maps histone methylation marks genome-wide without the bias of probes and with much higher sensitivity.

As evidence of its utility, ChIP-seq experiments in human cells have shown that H3K4me3, H3K4me2, and H3K4me1 are deposited in a gradient about active gene promoters. Generally, H3K4me3 forms a narrow, symmetric peak on either side of the transcription start site (TSS), while H3K4me2 and H3K4me1 form sequentially broader distributions about H3K4me3 [25]. H3K4me1 is additionally found at distal regions acting as enhancers (Fig. 2b) [29]. ChIP-seq and related methods have further allowed the study of the shape of peaks across the genome, opening up the field to an understanding of how the distribution of different epigenetic marks is dynamically regulated and influences gene expression [30, 31]. These general categorizations, however, are being increasingly recognized as overly simplistic, and as will be discussed later, dynamic regulation of H3K4 methylation marks throughout the genome may occur more often than previously thought.

While antibody-based techniques have been widely implemented to characterize histone PTMs such as H3K4 methylation, these techniques are limited in a few key areas. Examples of these include antibody cross-reactivity with structurally similar histone PTMs and antibody-binding affinity that varies with chromatin environment [32]. Mass spectrometry (MS) is an alternative technique that alleviates some of the concerns in using antibody-based tools, though it loses information on genomic location of the identified histone PTMs. Shotgun proteomics, a bottom-up proteomics strategy, consists of proteolysis to form peptides followed by liquid chromatography–tandem MS (LC–MS/MS). Analyzed peptides can then be mapped back to the protein by comparing the obtained tandem mass spectra with theoretical tandem mass spectra generated from in silico digestion of a protein database [33]. To make this analysis easier, an approach called middle-down proteomics can be used, where larger peptide fragments are generated to limit the possibility of having multiple matching sites for a given peptide. This strategy allows for the measurement of relative changes in H3K4 methylation without the typical limitations of an immunoassay. An added benefit of using MS instead of an immunoassay is that MS allows for the detection of multiple, and potentially novel, PTMs at the same time, so H3K4 methylation level can be studied in connection with other marks occurring on the same H3 peptide fragment [34]. While MS is an improvement in many regard to immunoassays, the abundance of the PTM, its stability, the shift in molecular weight imparted by the PTM, and its effect on the ionization of the peptide must all be considered, as these factors will strongly influence the difficulty of using MS to characterize the PTM [33].

It is important to analyze the regulation of H3K4 methylation in subregions and within specific cell types in the brain when investigating its function in learning and memory. Studies have implemented subdissections to assess potential spatial variability of H3K4 methylation level. Moreover, applying fluorescence-activated cell sorting (FACS) to the isolated nuclei of dissociated brain tissue has allowed analysis of epigenetic marks in neurons separately from other cell types [35]. This has been used in combination with ChIP, allowing interrogation of this mark in a cell type-specific manner, both at select sites with quantitative PCR (qPCR) and globally with sequencing [36, 37]. Theoretically, FACS sorting would allow cell type-specific analysis of H3K4 methylation when combined with any of the above-mentioned techniques.

## Dynamic control of H3K4 methylation: the writers and erasers

H3K4 methylation status is controlled by a series of lysine methyltransferase (KMT) “writers” that deposit methyl groups and lysine demethylase (KDM) “erasers” that remove them. While the members of this enzymatic cadre have been largely characterized, the mechanisms governing site-specific methylation by any particular one and the processivity of such reactions remain understudied. What is clearer, however, is that histone methylation is a dynamic process, with loss of basal methyltransferase or demethylase activity resulting in a decrease or increase in H3K4 methylation level, respectively, throughout the genome [7, 9]. The structure and reactivity of such enzymes have been reviewed previously; however, we provide a brief summary of the relevant details below [38–40].

### The writers: H3K4 methyltransferases

**Fig. 3:**
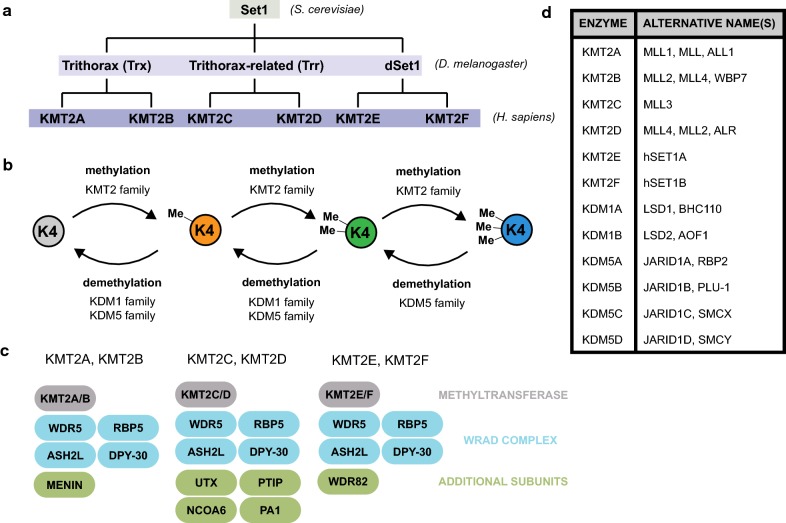
Enzymology of H3K4 methylation and demethylation. **a** Evolutionary tree diagram of H3K4 methyltransferases from yeast (*S. cerevisiae*) to flies (*D. melanogaster*) to humans (*H. sapiens*). **b** Diagram of H3K4 methylation and demethylation reaction pathways. Mono-, di-, and trimethylation reactions are carried out by all members of the KMT2 family. H3K4me1 and H3K4me2 demethylation reactions are carried out by KDM1 and KDM5 family members. The H3K4me3 demethylation reaction is carried out by KDM5 family members. **c** Bubble diagram of COMPASS-like complexes formed with each KMT2 family member. Methyltransferase subunits (gray) associate with a core WRAD complex comprised of WDR5, RBP5, ASH2L, and DPY-30 (blue). Additional subunits (green) associate with specific COMPASS-like complexes to influence target selection. **d** Table of H3K4 methyltransferase/demethylase enzymes and alternative names used in the literature

Like the modification itself, the H3K4 methylation machinery has been well conserved from yeast to humans. H3K4 methyltransferases were originally identified in yeast, which encode only one, Set1. Set1 operates in a complex called COMPASS, short for “Complex of Proteins Associated with Set1,” and is responsible for each mono-, di-, and trimethylation of H3K4 [41, 42]. *Drosophila*, on the other hand, express three Set1 homologs: dSet1, Trithorax (Trx), and Trithorax-related (Trr) [43]. Mammals, with correspondingly higher complexity, have six H3K4 methyltransferases in the Set1 homology cluster: KMT2A and KMT2B (homologs of *Drosophila* Trx), KMT2C and KMT2D (homologs of *Drosophila* Trr), and KMT2E and KMT2F (homologs of *Drosophila* dSet1) (Fig. 3a) [38].

Structurally, each of the mammalian H3K4 methyltransferases contains a 130–140 amino acid C-terminal catalytic Su(var)3-9, Enhancer-of-zester and Trithorax, or SET, domain that serves to catalyze methyl group transfer from S-adenosyl methionine (SAM) to the side chain lysine of H3K4 (Fig. 3b). Each methyltransferase catalytic subunit operates within a multiprotein COMPASS-like complex containing four shared components WDR5, RBP5, ASH2L, and DPY-30, together dubbed the “WRAD complex” [44]. Additional components associate with the COMPASS-like complexes of individual methyltransferases and may help direct the enzyme’s specificity for certain genomic areas. The tumor suppressor protein MENIN, for example, is a component of KMT2A and KMT2B complexes [45]. The H3K27 demethylase UTX, Pax Transactivation domain-Interacting Protein (PTIP), nuclear receptor coactivator (NCOA6), and PTIP-associated 1 (PA1) are each components of KMT2C and KMT2D complexes [46]. Lastly, WDR82 is a component of KMT2E and KMT2F complexes (Fig. 3c) [47]. In accordance with the formation of these different assemblies, human methyltransferases have been shown to harbor different targets as well. While KMT2E and KMT2F are thought to be responsible for global levels of H3K4 methylation, KMT2A–KMT2D have smaller and more specific sets of target genes, suggesting that their structural divergence reflects a corresponding functional divergence [47]. Given this extensive literature on KMT enzymology, historical differences in KMT nomenclature have arisen that convolute its interpretation. We report a list of alternative names for each KMT and KDM given recent clarification (Fig. 3d) [48].

### The erasers: H3K4 demethylases

Prior to the discovery of the first lysine demethylase, KDM1A (more commonly known as LSD1), in 2004, histone methylation was deemed by many a “permanent” epigenetic modification, reversible only by histone exchange [49]. After this seminal discovery, however, the identities and catalytic strategies of two major families of H3K4 demethylase enzymes were determined. An appreciation of demethylase function is now emerging, with the recognition that unrestricted H3K4 methylation can generate as much nuclear rancor as can overly restricted H3K4 methylation.

Humans have six H3K4 demethylases: KDM1 family members KDM1A and KDM1B, and KDM5 family members KDM5A, KDM5B, KDM5C, and KDM5D [40]. The KDM1 family enzymes remove methyl groups from only H3K4me2 and H3K4me1, while the KDM5 family enzymes remove methyl groups from each H3K4me3, H3K4me2, and H3K4me1—a substrate discrepancy that can be explained by different catalytic strategies (Fig. 3b). Structurally, KDM1 family enzymes are flavin adenine dinucleotide (FAD)-dependent and demethylate using an amine oxidase domain [49]. The intermediate species of such FAD-dependent reactions requires the presence of a lone pair of electrons on the side chain nitrogen of lysine in order to oxidize its single bond with the methyl group being removed. Therefore, by design, these enzymes cannot remove trimethylated lysines since the side chain nitrogen has no lone pair of electrons. The KDM5 family members, in contrast, harbor an N-terminal catalytic JumonjiC (JmjC) domain dependent on iron and alpha ketoglutarate cofactors [50]. The JmjC domain-containing enzymes do not require the formation of the nitrogen–carbon double bond and are therefore capable of demethylating trimethylated lysines. Like H3K4 methyltransferases, H3K4 demethylases also preferentially act at particular genomic locations; however, less is known about what factors guide such preferences for demethylases. For example, in the nervous system, KDM5C was shown to act predominantly at enhancer elements in mature neurons and modulate their basal activity by altering enhancer methylation levels [9]. Presumably, other demethylases act on other genomic regions to modulate their activity in a complex regulatory network. However, further studies are needed to determine these loci and what factors regulate them.

## H3K4 methylation as a quaternary system of transcriptional control

As for all histone modifications, there are two main mechanisms through which H3K4 methylation might function: First, H3K4 methylation might alter higher-order chromatin structure and second, methylated H3K4 might affect the binding of effector proteins to mediate downstream processes [51]. Since the electrostatic implications of histone methylation are not as drastic as those of other histone modifications such as acetylation and phosphorylation, more work has gone into identifying molecular interactors of methylated H3K4. In addition, earlier models of “electrostatic” mechanisms of histone regulation of DNA structure have largely been supplanted by more sophisticated effector-binding protein models over the last decade. Unmethylated H3K4, together with its products H3K4me1, H3K4me2, and H3K4me3, function together through these mechanisms, forming a quaternary system of transcriptional control.

The three methylated species—H3K4me1, H3K4me2, and H3K4me3—are summarized below in greater detail. Relatively less is known about unmethylated H3K4, though one function of this mark is permitting DNA methyltransferases to access their underlying sequence substrates. Structurally, an interface of de novo DNA methyltransferase DNMT3A and accessory protein DNMT3L binds unmethylated H3K4, an interaction that is inhibited by one or more methyl groups on H3K4 [52]. Genomic studies support this structural finding, with the observation that gene promoters lacking DNA methylation harbor higher levels of methylated H3K4 [53]. Thus, the presence of unmethylated H3K4 provides important information on the absence of activating H3K4 methyl marks, establishing transcriptionally silent heterochromatin and enabling DNA methylation at appropriate genomic loci.

### The monomethyl: H3K4me1

Monomethylation of H3K4 has garnered widespread attention as a chromatin signature of *enhancer elements*. Enhancers are regulatory elements comprised of a DNA segment that acts to increase the transcription of a gene. The original, and arguably stringent, definition of enhancers describes these elements as position independent, meaning that if the DNA segment is moved to different positions (upstream, downstream, and flipping the 5′ to 3′ orientation) relative to the gene it regulates, it will still retain its ability to activate transcription [54]. Enhancers often regulate tissue-specific gene expression and can come into physical contact with the promoters they regulate by folding the DNA into a loop [55].

The idea that H3K4me1 is an identifying mark of enhancers arose in the late 2000s, when Heintzman et al. reported a strong and broad enrichment of H3K4me1, in the absence of H3K4me3, at putative enhancers as defined by histone acetyltransferase p300 binding in HeLa cells [29]. Following this, a number of studies have used H3K4me1 profiles in conjunction with other chromatin marks and protein-binding sites to globally identify and predict cell type-specific enhancers [56, 57]. In the nervous system, for example, twelve thousand activity-regulated enhancers were identified in mouse cortical neurons using a combination of p300-binding profiles, H3K4me1 profiles, and location from TSSs as criteria [58]. Importantly, H3K4me1 levels at enhancers were shown to correlate with cell type-specific gene expression patterns, providing support of such functional designation [59]. Efforts to further stratify enhancers by activity level focused on other chromatin marks and proteins located at H3K4me1-enriched regions. In this regard, acetylation of H3K27 (H3K27ac) became recognized as a key distinguisher between active enhancers and poised enhancers, when present and absent at H3K4me1-enriched regions, respectively [60]. Functionally, active enhancers are those engaged in increasing the transcription of the target gene, while poised enhancers are those not currently engaged but which harbor the potential to be activated during cellular differentiation or in response to external stimuli.

While the above studies are globally correlative, they provide little information as to a causal role for H3K4me1 at enhancers. However, recent reports have begun to shed light on this matter. One study by Local et al. for example, took a proteomics approach to identify H3K4me1-associated proteins in mouse embryonic stem cells (mESCs) [61]. Interestingly, these authors found an association of the chromatin remodeling complex BAF with H3K4me1, positing that H3K4me1 facilitates binding of this complex and potentially others to enhancers. Supporting this idea, they additionally found that decreased binding of BAF components occurred at loci exhibiting H3K4me1 loss with catalytically dead versions of KMT2C and KMT2D. While this work furthers an understanding of H3K4me1’s potential interactome, it does not provide a clear role for H3K4me1 in mediating enhancer activity. More studies are needed, for example, to clarify the conflicted literature on the catalytic and non-catalytic functions of monomethyltransferases at enhancers. Another important consideration in the context of the brain is that the chromatin landscape in mESCs may not function as it would in a differentiated neuron. Further work in these differentiated cells is necessary to understand potential differences between such systems.

### The dimethyl: H3K4me2

While there are fewer reports on H3K4me2 than H3K4me1 or H3K4me3, this mark has been shown to be important in a phenomenon called *transcriptional memory*, defined as a change in rate and/or level of transcriptional induction at a gene due to prior transcriptional activity [62]. One of the first models characterized was inositol starvation in budding yeast, which produces transcriptional memory at the *INO1* gene encoding inositol-1-phosphate synthase [63]. In this model, transcriptional memory involves movement of the gene to the nuclear periphery and assembly of poised RNA polymerase II (Pol II) preinitiation complexes. Interestingly, work in both yeast and human immune cells have shown that H3K4me2 persists at genes after rapid transcriptional induction, even after H3K4me3 levels have returned to baseline [64, 65]. Additionally, loss of Set1 in yeast disrupts peripheral localization, suggesting that H3K4 methylation is necessary for transcriptional memory [65]. The mechanism of such a hypothesis was recently investigated by D’Urso et al. in the *INO1* model [66]. These authors found that H3K4me2 is necessary for the establishment of poised Pol II, directly linking this mark with transcriptional regulation. Further, they found that the Set1/COMPASS complex is remodeled to lack subunit Spp1 during transcriptional memory, making it incapable of H3K4 trimethylation, plausibly to promote dimethylation. Retained H3K4me2 at *INO1* recruits Set3, a member of the SET3C HDAC complex the authors postulate may be involved in excluding demethylases or recruiting remodeled Set1/COMPASS as a mechanism of H3K4me2 self-propagation. While there has been little corresponding mechanistic work in mammalian systems, the conservation of H3K4 methylation machinery suggests that there may be conserved regulatory mechanisms as well. For example, instead of COMPASS complex remodeling, mammalian cells may take advantage of different methyltransferases. Regardless, these findings in yeast provide a basis for further investigation in more complex systems.

### The trimethyl: H3K4me3

Since the early 2000s, the presence of H3K4me3 has been correlated with transcriptionally active promoters of genes, and H3K4me3 levels positively correlate with gene expression levels [25, 67]. H3K4me3 has been implicated in a number of nuclear processes, including Pol II-mediated transcription, pre-mRNA splicing, DNA recombination, and DNA repair [68–71]. In vitro transcription studies that allow absolute control over the amount of H3K4 methylation during transcription have revealed that increasing H3K4 methylation increases the transcriptional output of a gene [72].

Studying H3K4 methylation in vivo is more complicated due to difficulties with manipulating H3K4me3 without affecting other H3K4-methylated species or preventing potential confounds of non-histone targets of H3K4 regulatory machinery. Secondary effects caused by these manipulations are also hard to rule out. Indeed, a multitude of proteins have been shown to bind H3K4me3, including chromatin remodeling complexes such as CHD1, NURF, and tumor suppressors such as ING2 [73–77]. The recruitment of transcriptional suppressors suggests that H3K4me3 may also mediate gene repression or that transcriptional activation by H3K4me3 can be dampened through a negative feedback loop. These experimental complexities have prevented a unifying understanding of H3K4me3 in vivo. Under particular controversy is whether or not H3K4me3 is instructive for Pol II-mediated transcription in vivo, a question that has gone unanswered for almost two decades despite significant research efforts [78].

In support of the idea that H3K4me3 promotes transcription, H3K4me3 methyltransferase KMT2A was shown to interact with Pol II in mammalian cells, and loss of KMT2A function was shown to result in abnormally distributed Pol II, suggesting an intimate interaction between H3K4 methylation and transcriptional machineries [79]. Further, H3K4me3 itself was shown to recruit basal transcription factor TFIID to active genes via interactions with TAF3, as well as stimulate preinitiation complex (PIC) formation at p53-dependent promoters [72]. These findings are supported by studies conducted in neurons, as the magnitude of H3K4me3 gain at promoters in *Kdm5c* knockout animals correlated highly with the change in transcript levels, suggesting that degree of H3K4me3 increase predicts transcriptional upregulation [9]. Together, these studies are indicative of H3K4me3 binding and activating components of the transcriptional machinery, in a manner that explains correlation with transcript levels.

On the contrary, work across a variety of model organisms has indicated that H3K4me3 may not be vital for basic transcriptional programs. For example, in a biochemical reconstitution of elongation machinery in yeast, H3K4 methylation was shown to have no added effect on level of transcription [80]. However, the H3K4 methyltransferase activity in this assay was derived from immunoprecipitation of tagged ASH2 in HEK293 nuclear extracts, which may not be reflective of endogenous H3K4 methyltransferase complexes. Additionally, these experiments could not account for potential interactions with chromatin remodeling complexes such as CHD1, also known to bind methylated H3K4. Furthering the idea of a transcriptionally uninstructive role in flies, Howe et al. mutated all 48 copies of canonical and variant histone H3 genes in *Drosophila* cells to encode an amino acid that cannot be methylated in the place of lysine K4 [81]. These authors found mutated cells to be smaller and less proliferative than wild-type cells, but still able to produce targets of developmental signaling pathways, suggesting that H3K4 methylation is not strictly required for global transcription in these cells. Additionally, in a mammalian system, specific depletion of H3K4me3 via CxxC finger protein 1 (CFP1) deficiency led to only minor changes in the transcriptome of mouse ESCs as detected by microarrays, Pol II ChIP-seq, and GRO-seq, again suggesting that H3K4me3 is not integral for genome-wide transcriptional patterns [82]. It is still possible, though, that residual levels of H3K4me3 after CFP1 loss in these experiments could have been sufficient to play any role of H3K4me3 in transcription. Taken together, these studies generate two models for a potentially non-instructive role of H3K4me3 in transcription that still account for the widely replicated finding of a positive correlation with transcript levels, described in a recent review by Howe et al. [78]. First, H3K4me3 could be deposited as a result of transcription. Indeed, several studies have found that the transcriptional machinery is able to recruit H3K4 methyltransferases, and H3K4 methylation may be dependent on events occurring with transcription [83]. Second, H3K4me3 deposition could co-occur with transcription but remain independent of it—e.g., the factors regulating Pol II recruitment may also regulate recruitment of H3K4 methylation machinery. Indeed, in mESCs it has been shown that H3K4me3 can be deposited at CpG islands without surrounding promoter sequences in a CFP1-dependent manner and without the change in surrounding transcription levels [84]. Lack of transcriptional machinery in these regions could also suggest that H3K4me3 alone is not sufficient to initiate transcription. Overall, while these bodies of the literature supporting transcriptionally instructive and uninstructive roles of H3K4me3 are clearly discrepant, they demonstrate the many still-unanswered questions regarding this highly conserved chromatin mark. Furthermore, they provide support of the need for future investigation into the specificity of these results to each model system.

Challenging conventional notions of H3K4me3 and H3K4me1 as promoter- and enhancer-specific marks, respectively, several groups have recently reported H3K4me3 at highly active enhancers [85, 86]. The Adelman lab, for example, demonstrated that the ratio of H3K4me3 to H3K4me1 signal is positively correlated with enhancer strength, suggesting that this mark should not be ignored as an identifier of this type of regulatory element [86]. What remains to be resolved is whether the ChIP-seq signal observed is coming from modifications of the histones encasing the DNA of the enhancer sequence itself, or whether these highly active enhancers, which are known to loop to promoters, have such strong interactions with trimethylated promoters in 3D space that they get pulled down along with their associated promoters. Indeed, H3K4me1 signal, although slightly reduced at some of the most active enhancers, is often also seen at the same enhancers marked with H3K4me3, despite being mutually exclusive [85]. More work is required to distinguish these possibilities.

## H3K4me3 broad domains: a novel epigenetic signature

**Fig. 4:**
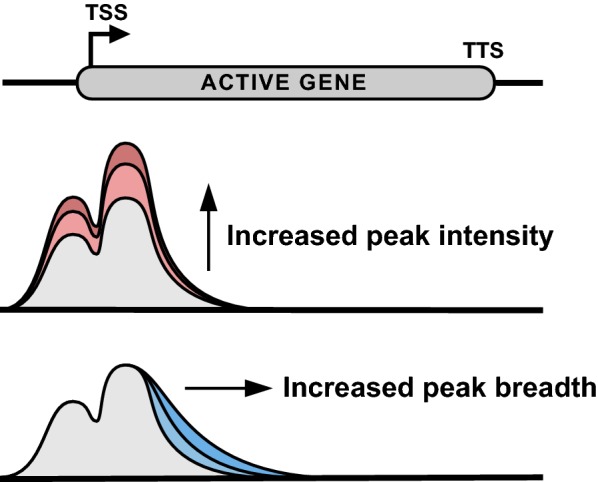
Intensity and breadth are distinct attributes of H3K4me3 peaks. H3K4me3 peaks are typically found upstream and downstream of the TSSs of active genes (TTS = transcription termination site). Peak intensity describes the level of H3K4me3 enrichment normalized to peak width. Peak breadth describes the width of H3K4me3 enrichment

As discussed above, the majority of H3K4me3 is distributed, in an approximately 1-kilobase footprint, symmetrically about the TSSs of active genes. However, recent work has identified a small subset of genes exhibiting an H3K4me3 distribution extending further downstream into the gene body—exhibiting increased H3K4me3 peak breadth, as opposed to increased H3K4me3 intensity (Fig. 4) [87]. This subset of H3K4me3-enriched regions has been variously called “broad domains” or “buffer domains,” the latter in reference to a proposed function of “buffering” against spurious bursts of transcription. This review will refer to this subset of regions as “H3K4me3 broad domains,” as their function has not yet been unambiguously determined. H3K4me3 broad domains are reviewed briefly below to document what is known about this new and interesting epigenetic signature.

In 2014, Benayoun et al. became the first group to extensively characterize H3K4me3 broad domains in a meta-analysis of H3K4me3 ChIP-seq data across a multitude of tissues and cell types [87]. As most H3K4me3 broad domains were found near promoter regions, the authors annotated the top 5% broadest H3K4me3 domains to genes with the closest TSS. Surprisingly, they found that this gene set was uniquely enriched for molecular elements necessary for cell type-specific features and factors required to establish cell lineage. The broadest domains of other activating epigenetic marks at promoters, such as H3K27ac, showed no such enrichment.

A report published not long after further parsed out the types of genes marked by H3K4me3 broad domains [88]. In ranking and dividing H3K4me3 broad domains into nine groups based on breadth conservation across 40 different cell types, Chen et al. found that the most conserved H3K4me3 broad domains were specifically enriched for tumor suppressor genes. These authors hypothesized that conserved H3K4me3 broad domains originate from embryonic stem cells and function in a high percentage of somatic cell types. Non-conserved H3K4me3 broad domains were found to be cell type-specific, suggesting that somatic cells acquire various additional H3K4me3 broad domains during their individual developmental trajectories.

The first study to implicate a specific histone methyltransferase in maintaining H3K4me3 broad domains was published recently by Dhar et al., showing that brain-specific loss of KMT2D led to decreased H3K4me3 levels at H3K4me3 broad domains and decreased expression of marked genes, with no apparent changes to genes marked by sharp H3K4me3 peaks [89]. Several other studies have examined the regulatory machinery that may be secondarily involved in maintaining broad H3K4me3 about promoters. Knockdown of Polycomb Repressive Complex 2 (PRC2)-associated factor Elongin BC and Polycomb Repressive Complex 2-associated protein (EPOP), for example, was found to promote narrowing of H3K4me3 about the TSSs of active genes [90]. Specifically at H3K4me3 broad domains, this loss of EPOP led to decreased H3K4me3 as well as decreased Pol II downstream of the TSS, suggesting that EPOP and H3K4me3 may be important in mediating release of Pol II from its elongating stage. Consistent with a potential role in transcriptional elongation, H3K4me3 broad domains exhibit high levels of positive transcription elongation factor *b* (P-TEFb) and serine-2-phosphorylated Pol II [87]. Further, the epigenetic mark H3K79me2, which has been shown to be important for the early phase of transcriptional elongation, shows strong signals at H3K4me3 broad domains relative to non-broad domains [88].

Despite these findings, the overall output of H3K4me3 broad domains within transcription remains controversial. As the term “buffer domain” implies, these domains have been associated with decreased transcriptional variance, suggesting that they may mark genes requiring high transcriptional fidelity [87]. However, other studies have demonstrated a significant correlation of H3K4me3 breadth with gene expression, suggesting, alternatively, that broad H3K4me3 promotes expression rather than maintains it at a constant rate [88]. To address these controversies, studies directly perturbing H3K4me3 breadth are much needed.

Though not yet widely studied in the central nervous system (CNS), one group has characterized H3K4me3 broad domains in human prefrontal cortex neurons from postmortem tissue, finding that ~ 120 domains, marking genes predominantly related to dopaminergic and glutamatergic signaling, were highly conserved across cortical neurons from other species [91]. This finding gives credence to the idea that H3K4me3 breadth provides a conserved function at genes important for cell identity, in neurons as is seen in other cell types. However, the interesting question remains as to whether H3K4me3 breadth undergoes dynamic regulation with neuronal activation or inhibition, as the intensity profiles of H3K4me3 itself and other epigenetic marks have been shown to undergo, discussed in the next section.

## H3K4 methylation in molecular mechanisms of memory

**Fig. 5:**
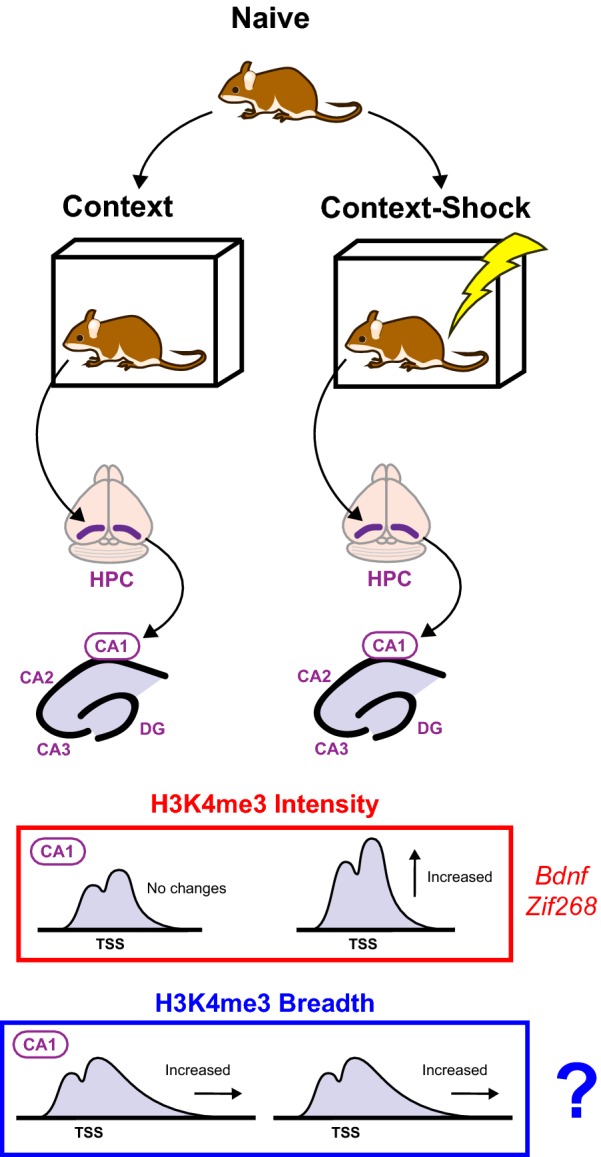
H3K4 methylation changes during CFC. In the CFC paradigm, naive mice are subjected to either a novel context (Context) or this novel context and an electric foot shock (Context–Shock) to precipitate formation of contextual and associative memories, respectively. Gupta et al. [5] dissected out the CA1 region of the hippocampus from these mice and subjected the tissues to ChIP-qPCR using primers to the promoters of learning genes. Increases in H3K4me3 intensity levels were observed at *Bdnf* and *Zif268* promoters in Context–Shock mice. However, it is currently unknown whether or not H3K4me3 breadth undergoes similar changes at learning-related genes in this paradigm

While previous work had suggested that H3K4 methylation was involved in regulating neuronal memory formation, the first direct evidence of this was published by Gupta et al. in 2010 [5]. This group found that, in trained mice that went through a contextual fear conditioning (CFC) paradigm where they received a foot shock associated with a new environment, or context, bulk levels of H3K4me3, as well as H3K4me3 at the promoters of specific memory-related genes *Zif268* and *Bdnf*, were elevated 1 h after associative learning in the HPC (Fig. 5). However, this effect did not persist to 24 h, suggesting that the action of a demethylase was responsible for its removal. These results demonstrated that trimethylation of H3K4 is dynamically regulated—seemingly transiently so—after associative memory formation.

Recent findings in rats have further shown that changes in H3K4me3 occur with memory retrieval. In the CA1 region of the HPC, a subregion providing critical outputs of the hippocampal memory circuitry, global levels of H3K4me3 were increased when these animals were given cues associated with a prior foot shock, compared to animals that had previously received a shock and were not presented with these cues [11]. This shows that increases in H3K4me3 occur with remembrance of a memory, not just as a response to sensory inputs causing the memory to be encoded. Furthermore, knockdown of methyltransferase *Kmt2a* in the CA1 after training reduced H3K4me3 levels and prevented retrieval of this memory, demonstrating a causal link between H3K4me3 and remembrance. Interestingly, ChIP-qPCR studies found that H3K4me3 levels increase within downstream coding regions of immediate early genes (IEGs) *Fos* and *Npas4*, not at their promoters, suggesting that the breadth of the H3K4me3 peak, not the intensity, was responsible for this global increase. Remarkably, H3K4me3 could be stimulated upon presentation of the fear-associated cue in the gene body of *Fos* even 30 days after the shock in anterior cingulate cortex, a brain region associated with memory storage.

In addition to studies of H3K4me3 level with memory formation and retrieval, knockout of H3K4 methyltransferases has also yielded insight into the importance of H3K4 methylation in learning and memory. Knockout of methyltransferase *Kmt2a* in the prefrontal cortex (PFC) of adult mice was found to cause significant deficits in spatial working memory compared to control animals, as assayed by T-maze and radial arm maze and in accordance with the PFC’s documented role in short-term memory [6]. These mice also exhibited an anxious phenotype and impaired nest building abilities, potential indicators of decreased social cognition. These behavioral impairments mirrored molecular impairments, as *Kmt2a* loss in the PFC led to a decrease in baseline expression of IEG *Arc*, as well as its blunted induction with radial arm maze. This builds upon the notion that H3K4 methyltransferases are important in maintaining proper cognition and permitting formation of new memories.

The above behavioral studies of mice lacking *Kmt2a* are complemented by electrophysiological and transcriptomics work published by Shen et al. in 2016. This group found that mice harboring *Kmt2a* knockout in postnatal forebrain demonstrated near-complete absence of spike-timing-dependent long-term potentiation (LTP) in ventral striatum, a type of synaptic plasticity integral for modulation of striatal circuitry [10]. Additionally, RNA sequencing of the striatum of these *Kmt2a*-deficient mice determined differential expression of a relatively small set of 262 genes including a number with a critical role in cognition, such as serotonin receptor *5*-*Htr2a* and nicotinic acetylcholine receptor *Chrna6*. Together, these findings implicate *Kmt2a* in a discrete synaptic-level function important for learning and memory.

In another example of brain-specific loss of a histone methyltransferase, conditional knockout of methyltransferase *Kmt2b* in mouse excitatory forebrain neurons was shown to cause performance deficits in the HPC-dependent learning paradigms novel object recognition, CFC, and Morris water maze [7]. Compared to wild-type animals, these conditional knockout mice differentially expressed a surprisingly small number (161) of genes in the dorsal dentate gyrus, the majority of which were downregulated. ChIP studies revealed that these downregulated genes harbored lower levels of H3K4me2 and H3K4me3 near their promoter regions, suggesting that KMT2B mediates H3K4 di- and trimethylation at these locations in a manner critical for memory formation.

While this barrage of data supporting a role for H3K4 methylation regulation in the mature nervous system is exciting, so too is emerging evidence that such regulatory enzymes may play non-redundant roles in mediating H3K4 methylation at gene targets. Kerimoglu et al. recently compared genes dysregulated in conditional forebrain *Kmt2a* and *Kmt2b* knockout mice, showing that these two gene sets overlap very little and suggesting that these two methyltransferases control largely distinct genomic regions [8]. Such a result reveals that different methyltransferases may be involved in unique molecular pathways leading to neuronal plasticity and memory formation. This observation suggests that a rich complexity of roles for H3K4 methylation might await discovery regarding its function in the CNS.

Importantly, the above-mentioned studies on H3K4 provide early investigation into a neuroepigenetic model of memory formation. However, further work is needed to resolve the spatial and temporal distributions of activity-dependent epigenetic modifications. For example, nearly all of the data reported here are from subregions of the hippocampus hours after memory formation, which reflects only a short period of time in a single brain region. It would be interesting to profile this mark at longer time points and in brain regions involved in memory storage to see whether similar changes are seen. Additionally, these studies do not profile H3K4 methylation changes in the set of neurons that are involved in forming the memory, known as engram cells, and thus, their results are reflective of engram and non-engram neurons undergoing potentially different transcriptional changes with memory. Moreover, engrams, often defined by expression of specific immediate early genes following a learning event, may inadequately represent different cell types exhibiting distinct transcriptional changes, necessitating broad experimental designs to truly link cell type-specific epigenetic changes with learning-induced transcription [92]. Though engram cells comprise a small proportion of neurons in the brain, making them technically challenging to isolate, approaches combining cell sorting with sequencing may elicit a better understanding of these cell type-specific changes and shed greater light on our understanding of neuroepigenetic involvement in memory formation.

## H3K4 methylation in cognitive disease

Clinical data in humans have supported the idea that H3K4 methylation is important for cognition [93–96]. One line of evidence stems from the observation that mutations in components of the H3K4 regulatory machinery cause a series of rare neurodevelopmental disorders involving intellectual disability (ID) and are associated with more complex diseases such as autism spectrum disorder (ASD) and schizophrenia (Table 1). A second line of evidence arises from the finding of altered H3K4 methylation patterns in postmortem brain tissue of patients with ASD. Further, research in rodents has shown that H3K4 methyltransferases and demethylases are important for reward-based learning, implicating H3K4 methylation in substance-related and addictive disorders. Each of these is discussed below in further detail.

**Table 1:** H3K4 methylation machinery implicated in cognitive disease

Enzyme	Associated cognitive disease	OMIM #	Mutation type	References
KMT2A	Wiedemann–Steiner syndrome	605130	Predicted LOF	[12, 97–99]
KMT2B	Dystonia 28	617284	Predicted LOF	[13, 100]
KMT2C	Kleefstra syndrome 2	617768	Predicted LOF	[14, 98, 101–103]
KMT2D	Kabuki syndrome 1	147920	Predicted LOF	[15, 104]
KMT2E	ASD	n/a	Predicted LOF	[16]
KMT2F	Schizophrenia	n/a	Predicted LOF	[19, 105]
KDM1A	CPRF	616728	Unknown	[17, 106]
KDM5A	ID	n/a	Predicted LOF	[107]
KDM5B	ASD, ID	n/a	Unknown	[97, 102, 103, 108]
KDM5C	Claes–Jensen syndrome, ASD	300534	Predicted LOF	[18, 109]

Associated disease(s) are listed in the second column. Online Mendelian Inheritance in Man (OMIM) # reported for each disease in the third column. Mutation type, if predicted or unknown, reported in the fourth column (*LOF* loss of function). References contributing significantly to an understanding of disease etiology or clinical manifestations are listed in the fifth column

With the advent and clinical translation of technologies such as whole-exome sequencing, it has become financially feasible to identify causative mutations in patients with rare diseases. For example, the etiology of Wiedemann–Steiner syndrome (OMIM #605130) was discovered in 2012 through whole-exome sequencing of six individuals with an internally consistent set of phenotypic characteristics [12]. Caused by heterozygous mutations in *KMT2A* that are predicted to be loss of function, Wiedemann–Steiner syndrome involves mild-moderate ID, behavioral difficulties, and hypertrichosis cubiti [97–99]. Mutations in its sister methyltransferase *KMT2B*, however, cause a hyperkinetic movement disorder called dystonia 28 (OMIM #617284), which is associated with mild-moderate cognitive impairment in a subset of patients [13, 100]. Other diseases attributed to mutations in H3K4 methyltransferases include Kleefstra syndrome 2 (OMIM #617768), caused by mutations in *KMT2C*, and Kabuki syndrome 1 (OMIM #147920), caused by mutations in *KMT2D* [14, 15, 98, 101, 104]. These syndromes are both neurodevelopmental disorders with variable degrees of ID and are individually associated with additional medical conditions including motor dysfunction, epilepsy, and congenital defects. In addition, while not tied to a specific syndrome, H3K4 methyltransferases *KMT2E* and *KMT2F* have been associated with the behaviorally complex diseases ASD and schizophrenia, respectively [16, 19, 105].

Along with H3K4 methyltransferases, H3K4 demethylases have also been associated with cognitive disease. The etiology of X-linked Claes–Jensen syndrome (OMIM #300534) was discovered in 2005 to be mutations in *KDM5C* [18]. Patients with Claes–Jensen exhibit ID, short stature, hyperreflexia, and distinctive facial features. Interestingly, one patient with an identified hemizygous mutation in *KDM5C* lacked the behavioral and facial characteristics of Claes–Jensen syndrome and was instead diagnosed with ASD [109]. This suggests clinical variability may result from mutations in different locations of the *KDM5C* gene. Other demethylases implicated in cognitive dysfunction include KDM1A, mutations of which cause the rare syndrome CPRF (OMIM #616728), named for cleft palate, psychomotor retardation, and distinctive facial features that characterize it [17, 106]. Additionally, mutations in *KDM5A* have been reported in autosomal recessive ID, while mutations in *KDM5B* have been linked to cases of both ASD and ID [97, 102, 103, 107, 108].

Apart from genetic disruption of H3K4 methylation machinery, aberrant H3K4 methylation patterns, particularly involving H3K4me3 breadth, have also been implicated in other complex neurologic and psychiatric illnesses. For example, genes harboring H3K4me3 broad domains are preferentially downregulated in mouse models of Huntington’s disease [110]. Further, abnormal “spreading” of H3K4me3 into gene bodies has been shown to occur in some cases of ASD [111].

The function of H3K4 methyltransferases and demethylases has also been shown to facilitate addictive behaviors in mice. As is the case for many studies of reward-based learning, a rodent model of addiction was used to simulate maladaptive memory formation involving the context of a rewarding drug of abuse. Specifically, Aguilar-Valles et al. used a model of methamphetamine conditioned place preference (CPP) in mice to study how H3K4 methylation is involved in forming memories of this drug of abuse with the context in which it is received [4]. They focused on the nucleus accumbens (NAc), a brain region in the ventral striatum which receives inputs from the ventral tegmentum, medial PFC, hippocampus, and amygdala and is known be to an integral center of reward-based learning. Interestingly, local knockdown of methyltransferase *Kmt2a* in the NAc prior to training led to decreased bulk H3K4me3 in the NAc and decreased CPP. In contrast, knockdown of demethylase *Kdm5c* had no effect on memory formation prior to training; however, its knockdown following training led to increased bulk H3K4me3 in the NAc and prevention of memory expression. These findings implicate the H3K4 methylation machinery as integral to proper reward-based memory formation and retrieval. Though not yet studied in humans, this work suggests that disrupting the H3K4 methylation machinery at different points of the addiction cycle may provide therapeutic benefit.

## Drugging the H3K4 methylome

It should be noted that, despite this diagnostic diaspora, nearly no therapeutics are available to such patients for cognitive dysfunction. For example, treatment for the neurodevelopmental disorders ID and ASD are largely limited to behavioral interventions and symptom management. While individually rare, together mutations in chromatin modifiers, particularly modifiers of H3K4 methylation, constitute a significant portion of cognitive disease, and therapeutic development for these diseases would fill a vast clinical gap. It would certainly be worth exploring whether ID from loss-of-function mutations in H3K4 methyltransferases could be improved by inhibiting demethylases to balance the level of H3K4 methylation, and vice versa for mutations in demethylases. Thus far, effort has gone into developing therapeutics targeting H3K4 methylation machinery in the context of other diseases but not ID. Peptidomimetic inhibitors of the KMT2A/WDR5 interaction domain, for example, have shown efficacy in slowing growth of leukemia cells in vitro [112]. Further, a series of studies have developed inhibitors of the KDM5 family members; however, limited potency and selectivity remain a challenge to their clinical translation [113, 114]. Promisingly, three drugs targeting H3K4 demethylases have reached clinical trials: KDM1A inhibitor ORY-1001 for acute myeloid leukemia and small cell lung cancer, KDM1A inhibitor GSK2879552 for myelodysplastic syndromes, and KDM5 inhibitor GS-5801 for chronic hepatitis B infection. While further work is needed to develop more specific drugs, these compounds provide a conceptual, if not structural, foundation. Given the non-redundant roles of H3K4 methylation and demethylation machineries, especially within the KMT family, the design of inhibitors has the potential to very specifically target subsets of genes.

A major consideration in drugging the methylome, however, is onset of pathology causing cognitive impairment—whether cognitive deficits are due to impaired neurodevelopment or impaired plasticity in the developed brain, given that H3K4 methylation is involved in both processes. Several groups have tried to parse out these mechanisms with conditional knockout mouse models. Scandaglia et al., for example, generated whole-body and inducible forebrain neuron-restricted *Kdm5c* knockout mice to dissect cognitive phenotypes arising from developmental loss and adult loss of this H3K4 demethylase [9]. While adult loss of *Kdm5c* in forebrain neurons did elicit a specific spatial learning delay in hidden platform and reversal phases of Morris water maze, developmental whole-body loss of *Kdm5c* led to a more severe cognitive phenotype including memory deficits in CFC and increased impulsivity. These data suggest that a large portion of, but not all, cognitive impairment in whole-body knockout mice arises from impaired neurodevelopment. However, it is still possible that adult loss of *Kdm5c* in other brain regions or cell types may contribute to greater cognitive impairment than that seen in forebrain neurons. In a similar approach, conditional forebrain neuron *Kmt2a* and *Kmt2b* knockout mouse models have been generated to explore adult loss of these methyltransferases [7, 8]. Whole-body knockout of either of these enzymes is embryonic lethal, necessitating such conditional models [115, 116]. However, adult forebrain neuron loss of *Kmt2a* or *Kmt2b* leads to impaired hippocampus-dependent memory formation. Thus, loss of these methyltransferases during adulthood is less severe than loss during development, but both are cognitively debilitating. Together, these studies confirm the importance of differentiating behavioral outcomes of dysregulated H3K4 methylation during development and in the developed brain. Whether or not cognitive phenotypes arising from impaired neurodevelopment are therapeutically irrevocable, however, remains to be seen. While there are no such studies for mutations in H3K4 methylation machinery, work in other genetic disorders of chromatin regulators involved in neurodevelopment, such as MeCP2 in Rett syndrome, have shown that adult treatment can indeed reverse an aggregate phenotype [117]. Thus, though preclinical studies and clinical trials of H3K4 modifier inhibitors may be far off, knowledge of this mechanistic distinction may help inform treatment options and expectations for patients and their families in the future.

## Conclusions

H3K4 methylation has gained widespread recognition as a critical epigenetic modification in cellular development and differentiation, yet its role in the nervous system is only marginally understood. As detailed above, recent evidence has shown that loss of components of the H3K4 methylation machinery, along with the H3K4 methylation marks they confer, causes deficits in behavioral correlates of learning and memory. Furthermore, these findings are clinically recapitulated, as patients with deleterious mutations in these enzymes are often afflicted with ID, and, with the advent of genome-wide association studies of CNS disorders, one suspects additional roles for H3K4 methylation in CNS pathology are likely to be discovered. While this body of work buttresses the notion of a neuroepigenetic basis to cognition, it also mandates further mechanistic research. More studies are needed, for example, to parse out the specific molecular partners of each H3K4me1, H3K4me2, and H3K4me3 in vivo, as well as to clarify their individual antecedent roles, if any, in neuronal transcriptional regulation. A better understanding is needed, too, of how a multiplicity of epigenetic marks change in the nucleus of a cell with many diverse connections to permit synapse-specific modification that occurs with learning. Additionally, from a pharmacological standpoint, the development of targeted therapeutics for H3K4 methyltransferases and demethylases remains an open and worthwhile synthetic challenge. Applying these potential therapeutics to the study of cognitive disease will be informative as to whether they could be a viable avenue to provide benefit to populations with very few to no pharmacological treatment options. With these aims in mind, the study of H3K4 methylation portends a promising future line of investigation within neuroepigenetics.

## Abbreviations

ASD: autism spectrum disorder
CFC: contextual fear conditioning
CFP1: CxxC finger protein 1
ChIP: chromatin immunoprecipitation
CNS: central nervous system
COMPASS: Complex of Proteins Associated with Set1
CPP: conditioned place preference
CPRF: cleft palate, psychomotor retardation, and distinctive facial features
DNMT3A: DNA (cytosine-5)-methyltransferase 3A
EPOP: Elongin BC and Polycomb Repressive Complex 2-associated protein
FACS: fluorescence-activated cell sorting
FAD: flavin adenine dinucleotide
H3K27ac: histone H3 lysine K27 acetyl
H3K4: histone H3 lysine K4
H3K4me1: histone H3 lysine K4 monomethyl
H3K4me2: histone H3 lysine K4 dimethyl
H3K4me3: histone H3 lysine K4 trimethyl
H3K79me2: histone H3 lysine 79 dimethyl
HPC: hippocampus
ID: intellectual disability
IEG: immediate early gene
JmjC: JumonjiC
KDM: lysine demethylase
KMT: lysine methyltransferase
LC–MS/MS: liquid chromatography–tandem mass spectrometry
mESC: mouse embryonic stem cell
MS: mass spectrometry
NAc: nucleus accumbens
NCOA6: nuclear receptor coactivator 6
P-TEFb: positive transcription elongation factor b
PA1: PTIP-associated 1
PFC: prefrontal cortex
PIC: preinitiation complex
Pol II: RNA polymerase II
PRC2: Polycomb Repressive Complex 2
PTIP: Pax Transactivation domain-Interacting Protein
PTM: posttranslational modification
qPCR: quantitative PCR
SAM: S-adenosyl methionine
SET: Su(var)3-9, Enhancer-of-zester and Trithorax
Trr: Trithorax-related
Trx: Trithorax
TSS: transcription start site
TTS: transcription termination site
